# Commentary: Autonomic Modulation in Patients with Heart Failure Increases Beat-to-Beat Variability of Ventricular Action Potential Duration

**DOI:** 10.3389/fphys.2017.00459

**Published:** 2017-06-30

**Authors:** Valerie Y. H. Van Weperen, Marc A. Vos, Marcel A. G. Van der Heyden

**Affiliations:** Department of Medical Physiology, Division of Heart and Lungs, University Medical Center UtrechtUtrecht, Netherlands

**Keywords:** autonomous nervous system, heart failure, valsalva maneuver, action potential duration variability, beta-blocker, activation recovery interval

Porter et al. (2017) recently published an interesting paper in which they explored the *in vivo* effect of the sympathetic nervous system (SNS) on cardiac electrophysiological stability. In their study, heart failure (HF) patients treated by cardiac resynchronization therapy (CRT) increased their sympathetic tone by performing the Valsalva maneuver. Comparison of the electrophysiological consequences of this stimulation on and off treatment with bisoprolol, allowed assessment of the effect of SNS stimulation. Their results demonstrated a significant 53% increase of the beat-to-beat variability of activation recovery intervals (ARIs) in absence of bisoprolol, whereas presence of bisoprolol restrained this increase to 11%.

For many decades, hyperactivity of the SNS has been identified as a factor in the development of arrhythmias (Schwartz, 2014; Shen and Zipes, 2014). In addition, surgical sympathetic denervation of the heart has long been established as an effective anti-arrhythmic therapy. The first stellectomy dates back to 1916, when Jonnesco employed the intervention and successfully freed a patient of angina pectoris and recurrent episodes of ventricular arrhythmias (Jonnesco, 1921). Successive research increasingly elucidated the distressing effects of the SNS on the heart and increasingly deciphered the causal relationship between activity of the SNS, especially that of the left stellate ganglion (Schwartz and Stone, 1980), and arrhythmogenesis (Zhou et al., 2008). However, these perturbing sympathetic effects resulting in ventricular arrhythmias are only present in abnormal cardiac conditions, as in HF and/or long QT-syndrome patients (Rubart and Zipes, 2005).

The extent of this pro-arrhythmic contribution can be evaluated by assessing the electrophysiological stability of the heart. Beat-to-beat variability of, for example, ventricular repolarization or ARIs is an indicator of cardiac electrophysiological stability (Thomsen et al., 2004; Varkevisser et al., 2012). Repolarization robustness may be quantified by the short-term variability (STV), a parameter that represents the average deviation of *n* number of consecutive beats from the line of identity in a Poincaré plot. The electrophysiological disturbances caused by pro-arrhythmic factors, e.g., through decreasing the repolarization reserve, will progressively increase the beat-to-beat variability and thus also the STV. In their study, Porter et al. assessed this parameter to explore the effects of beta blockade on electrophysiological stability. However, in contrast to the standard, and more robust, calculation of STV using 30 consecutive beats (Thomsen et al., 2005), Porter et al. based their results on the ARI of 10 consecutive beats, probably forced by the short duration of the effects of the Valsalva maneuver. Reasonably, the use of less beats allows outliers to have a disproportionate effect on the STV, possibly giving rise to an overestimation of the anti-arrhythmic efficacy of beta-blocking drugs. In addition, ARI based calculations of STV are highly dependent on the morphology of the ARI. Hence, temporal inconsistencies in its morphology, for example in electrophysiological instable and/or challenging situations, might translate to a decreased reliability of the calculated STV (Oosterhoff et al., 2010).

Furthermore, Porter et al. might have underestimated and/or overlooked the influence of non-electrophysiological parameters on the beat-to-beat variability. As previously determined by Stams et al. (2016) mechanical effects, such as fluctuating preload, may independently cause significant increases in STV. Since the Valsalva maneuver induces a substantial variability in preload, one could argue that the observed increase in STV could, to some extent, be ascribed to these mechanical effects.

Neuromodulation as an anti-arrhythmic therapy has however not advanced to the level that it is much implemented in the clinic. An explanation could be found in the lower anti-arrhythmic efficacy of neuromodulating drugs, such as beta blockers, in comparison to more effective surgical denervation. Although there are no studies on this discrepancy, several hypotheses have been suggested.

First, the utilization of drugs is accompanied by limiting factors such as safety issues, pharmacokinetic, and dynamic characteristics, but also drug refractoriness in patients. In addition, most neuromodulatory drugs block a smaller spectrum of sympathetic input than a stellectomy may achieve. For example (selective) beta-blockers such as bisoprolol, which was used in the paper of Porter et al. (2017), does not affect beta 2- as well as alpha-adrenergic receptors, thereby leading to incomplete removal of sympathetic effects on the myocardium.

Second, interference with the postganglionic transmission of sympathetic stimuli, as most drugs do, in general induces upregulation of adrenergic receptors causing increased sensitivity to agonists (Haeusler, 1990). This adverse effect may not occur after stellectomy (Schwartz and Stone, 1982). This critical difference in cardiac response to either form of denervation probably underlies the difference in efficacy of pharmacological and surgical denervation therapies.

It is thus through simple yet cogent reasoning that one has to conclude that sympathetic interference may present a potent, yet undervalued, anti-arrhythmic strategy. Cardiac sympathetic denervation has already proven itself to be an effective treatment against ventricular arrhythmias in patients. Achieving as equally successful results through pharmacological sympathetic blockade remains a challenge. As presented by Porter et al. (2017), though successfully reducing the beat-to-beat variability in ARI, the sympathetic effects were not fully eliminated. Careful analysis of studies reporting a possible anti-arrhythmic effect of neuromodulatory drugs, such as presented in the paper by Porter et al. (2017), might contribute to finding an explanation for the variable efficacy of pharmacological sympathetic blockades in disengaging the heart from its sympathetic influence. Therefore, the results of Porter et al. (2017), may be instrumental in the development of new pharmacological anti-arrhythmic therapies.

## Author contributions

All authors listed have made a substantial, direct and intellectual contribution to the work, and approved it for publication.

### Conflict of interest statement

The authors declare that the research was conducted in the absence of any commercial or financial relationships that could be construed as a potential conflict of interest.
